# OpenMEEG: opensource software for quasistatic bioelectromagnetics

**DOI:** 10.1186/1475-925X-9-45

**Published:** 2010-09-06

**Authors:** Alexandre Gramfort, Théodore Papadopoulo, Emmanuel Olivi, Maureen Clerc

**Affiliations:** 1 Athena Project Team, INRIA Sophia Antipolis Méditerranée, France; 2 Parietal Project Team, INRIA Saclay Ile-de-France, France

## Abstract

**Background:**

Interpreting and controlling bioelectromagnetic phenomena require realistic physiological models and accurate numerical solvers. A semi-realistic model often used in practise is the piecewise constant conductivity model, for which only the interfaces have to be meshed. This simplified model makes it possible to use Boundary Element Methods. Unfortunately, most Boundary Element solutions are confronted with accuracy issues when the conductivity ratio between neighboring tissues is high, as for instance the scalp/skull conductivity ratio in electro-encephalography. To overcome this difficulty, we proposed a new method called the symmetric BEM, which is implemented in the OpenMEEG software. The aim of this paper is to present OpenMEEG, both from the theoretical and the practical point of view, and to compare its performances with other competing software packages.

**Methods:**

We have run a benchmark study in the field of electro- and magneto-encephalography, in order to compare the accuracy of OpenMEEG with other freely distributed forward solvers. We considered spherical models, for which analytical solutions exist, and we designed randomized meshes to assess the variability of the accuracy. Two measures were used to characterize the accuracy. the Relative Difference Measure and the Magnitude ratio. The comparisons were run, either with a constant number of mesh nodes, or a constant number of unknowns across methods. Computing times were also compared.

**Results:**

We observed more pronounced differences in accuracy in electroencephalography than in magnetoencephalography. The methods could be classified in three categories: the linear collocation methods, that run very fast but with low accuracy, the linear collocation methods with isolated skull approach for which the accuracy is improved, and OpenMEEG that clearly outperforms the others. As far as speed is concerned, OpenMEEG is on par with the other methods for a constant number of unknowns, and is hence faster for a prescribed accuracy level.

**Conclusions:**

This study clearly shows that OpenMEEG represents the state of the art for forward computations. Moreover, our software development strategies have made it handy to use and to integrate with other packages. The bioelectromagnetic research community should therefore be able to benefit from OpenMEEG with a limited development effort.

## Introduction

Many devices used in the clinical or the cognitive science domain perform electromagnetic measurements, or stimulation, on the human body. Devices measuring electric fields include electro-encephalograms (EEG), -cardiograms (ECG), -myograms (EMG), while magneto-encephalograms (MEG) or magneto-cardiograms (MCG) measure magnetic fields. Among stimulating devices, transcranial magnetic stimulation (TMS) uses magnetic coils to stimulate brain regions, while functional electric stimulation (FES) and electrical impedance tomography (EIT) impose an electric current or potential through contact electrodes.

To interpret and control the bioelectromagnetic phenomena involved with these devices, realistic physiological modeling is required, in terms of geometry and conductivity [1]. Accurate numerical solutions of the governing equations must be computed: obtaining the best accuracy possible for a given computational model is one of the goals of OpenMEEG, the opensource software package introduced in this article.

Electromagnetic propagation is governed by the Maxwell equations, coupling the electrical and magnetic fields. This coupling simplifies when the relevant frequencies are low enough for the quasistatic regime to hold. The electric potential is then governed by the law of electrostatics

(1) $$\nabla \cdot (\sigma \nabla V)=\nabla \cdot J^{\text{p}},$$

Where *σ *is the conductivity field, and **J**^p ^is a dipolar source distribution within the domain. When considering brain activations, it represents average postsynaptic currents within pyramidal cortical neurons. A boundary condition must be imposed, typically controlling the normal current on the domain boundary:

(2) σ∇V⋅n=j.

The electric potential can be computed independently from the magnetic field, by solving (1) with boundary condition (2). The magnetic field **B **depends both on the electric potential *V *and on the current source distribution **J**^p^, through the Biot and Savart law.

(3) $$B\text{(}r\text{)}=\frac{\mu_{0}}{4\pi }\int (J^{\text{p}}(r^{\prime })-\sigma \nabla V(r^{\prime }))\times \frac{r-r^{\prime }}{\|r\text{–}r^{\prime }{\|}^{3}}dr^{\prime },\;$$

written here in the case where *j *= 0 on the boundary.

A *forward problem *consists of simulating *V *and/or **B **when *σ*, **J**^p^, and boundary current *j *are known. The forward electro- magnetostatics problem is well-posed, in contrast to the far more difficult, ill-posed *inverse problem *of estimating *σ*, or **J**^p^, from partial boundary data. Still, obtaining an accurate solution for the forward problem is far from trivial. This paper presents a software package, OpenMEEG, that makes available to the community recent research efforts to improve the accuracy of forward solvers.

Forward solutions provide the relationship between the quantities of interest and the measurements. To obtain a good description of this relationship, one must model the sources, the conductivity, and the sensors.

**Figure 1:**
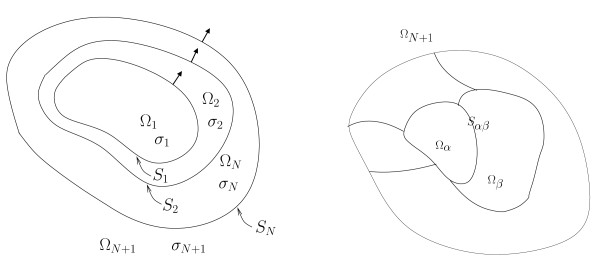
**Models for Boundary Elements Methods**. Boundary Elements are well-suited for piecewise constant isotropic conductivity models. OpenMEEG handles nested regions (left), and could in principle be extended to more general, disjoint regions (right) as presented in [8].

The choice of conductivity model is especially delicate because it strongly constrains the numerical solutions that can be used to solve the problem. In all generality, the conductivity field *σ *should be modeled as a tensor field, because composite tissues such as bone and fibrous tissues have an effective conductivity that is anisotropic. Realistic, anisotropic conductivity models can however be difficult to calibrate and handle: simpler, semi-realistic, conductivity models assign a different constant conductivity to each tissue, as depicted in Figure 1. There are three main types of conductivity models, and associated numerical methods:

1. if the conductivity field can be described using simple geometries (with axilinear, planar, cylindrical, spherical or ellipsoidal symmetry), *analytical methods *can be derived, and fast algorithms have been proposed that converge to the analytical solutions for EEG [2,3] and MEG [4];

2. for piecewise constant conductivity fields as in Figure 1, *Boundary Element Methods *(BEMs) can be applied, resulting in a simplified description of the geometry only on the boundaries [5];

3. general non-homogeneous and anisotropic conductivity fields are handled by *volumic methods*; Finite Element Methods and Finite Difference Methods belong to this category.

This paper deals with Boundary Element Methods, whose advantage over volumic methods is to use an economic representation of the conductivity field (a few conductivity parameters - one per tissue, and a few triangular meshes to represent the interfaces). Until recently, all Boundary Element Methods used in bio-electromagnetics were based on a Green representation theorem, involving operators called single- and double-layer potentials. In OpenMEEG the approach is to consider an extended version of the Green representation theorem [6], involving more operators, and leading to a new BEM formulation, called the Symmetric Boundary Element Method [7].

The structure of the paper is as follows: the applications targeted by OpenMEEG are first presented, then some mathematical aspects of the Symmetric BEM are explained (details are presented in an appendix). Then, to motivate the use of OpenMEEG, a comparison study with four other solvers in the context of EEG forward modeling is presented. The accuracy of the solvers is tested on multiple random sphere models. The accuracy of OpenMEEG is also assessed by numerical experiments for MEG forward modeling. Finally a section provides technical details on the OpenMEEG software package while giving sample code for using OpenMEEG via Python or via the Fieldtrip Matlab toolbox. A more complete presentation of the usage of OpenMEEG via a command line interface is available in Additional file 1.

### Target applications of OpenMEEG

The main purpose of OpenMEEG is to solve the forward problems arising in magneto- and electro-encephalography. OpenMEEG is therefore primarily developed in the field of brain research, but its development could directly benefit to other problems dealing with electromagnetic biosignals (see for example [8] in the context of electrocardiography). A geometrical model of nested meshes representing tissue interfaces must be provided to OpenMEEG, which does not perform any segmentation or meshing. OpenMEEG handles nested regions, and could in principle be extended to more general, disjoint regions [9]. The main output of a forward problem is a leadfield, i.e., the linear application relating sources at specific locations to sensor measurements. Although the principal target of OpenMEEG is magneto- and electro-encephalography, other types of bioelectromagnetic problems have also been handled with OpenMEEG: we hereforth describe its current scope.

**Electroencephalography (EEG) **is concerned with the variations of electric potential on the scalp, due to sources within the brain. At frequencies of interest, the quasistatic regime is valid, resulting in the electrostatics relation (1). The air surrounding the scalp is supposed non-conductive, hence the normal current vanishes on the scalp: *j *= 0 in boundary condition (2). The sources within the brain are represented by dipoles: in equation (1), the sources are $J^{\text{p}}=\vec{q}\delta_{p\;}$ where *p *is a dipole position and $\vec{q}$ the associated dipolar moment. OpenMEEG computes the electric potential and the normal current on each interface between two homogeneous tissues, due to electric sources within the brain. EEG sensors are electrodes, modeled in OpenMEEG as discrete positions on the scalp at which the potential can be measured (infinite impedance assumption). On these sensors, OpenMEEG computes the EEG leadfield, representing the linear relationship between source amplitude (for fixed position and orientation) and sensor values.

**Magnetoencephalography (MEG) **is concerned with magnetic fields produced by sources within the brain, which, like for EEG, are modeled as dipoles [10]. OpenMEEG makes use of the Biot and Savart relation (3) to compute the magnetic field, and hence requires the electric potential to be computed beforehand on all the interfaces [11]. Magnetometers or gradiometers can be modeled in OpenMEEG. Magnetometers are defined by their position and the direction of the field they measure. Gradiometers and more general sensors are handled by providing to OpenMEEG the positions, the orientations and the weights of integration points. For example with axial gradiometers present in CTF MEG systems, the forward field for a sensor is obtained by subtracting the two leadfields computed at the locations of two nearby magnetometers.

**Electrical Impedance Tomography (EIT) **infers characteristics of a conductive domain, by analyzing the potential resulting from the application of a current on the boundary. This method has been applied to calibrate the conductivity of EEG head models [12-14]. OpenMEEG computes the forward problem associated to EIT: given a conductive domain Ω defined by the interfaces between homogeneous regions, and their conductivity, and for a prescribed normal current *j*, OpenMEEG computes the potential *V *and the normal current *σ*∂_**n **_*V *on each interface by solving (1) for **J**^p ^= 0, and with boundary condition (2). The electrodes are modeled with a P0 approximation over the triangles of the scalp; the injected current is assumed constant over a triangle.

**Intracranial electric potentials **are measured in certain clinical settings, either on the surface (ElectroCorticography) or within the brain (intracranial EEG, or stereotaxic EEG). OpenMEEG is able to compute leadfields for such intracranial electrodes, in realistic head models.

**Functional Electrical Stimulation (FES) **provides a way to restore movement of paralyzed body regions by activating the efferent somatic axons. For this a current is applied to a nerve sheath, using specially designed electrodes. The precise location and time course of the applied current must be optimized in order to achieve the best selectivity, and to minimize the current intensity for a desired outcome. Optimizing the stimulation parameter in a realistic nerve model requires a forward model for FES. OpenMEEG provides such a tool, by combination of the concepts of EIT and of internal potential simulation (see section on the applications targeted by OpenMEEG) [15,16].

**Cortical Mapping **is an *inverse problem *that aims to recover the potential and the normal current occurring on the surface of the cortex (i.e., under the skull bone), given EEG measurements on electrodes [17]. A particularly elegant solution to this problem has been proposed with the symmetric BEM [18], making it possible e.g. to solve further source localization problems.

### Methods: implementation

OpenMEEG uses a Galerkin Boundary Element formulation, that jointly considers the electric potential and the normal current on each interface as unknowns of the problem. A P1 (piecewise linear) approximation is used for the electric potential, whereas the normal current $\sigma \frac{\partial V}{\partial n}$ is approximated with P0 (piecewise constant) boundary elements. The most intricate part of the implementation concerns the assembly of the system matrix, requiring singular kernel integration over triangles. Those are double integrals. The inner integrals are computed with analytical schemes [19], whereas the outer integrals are computed with a 7-point Gauss quadrature scheme [20]. An adaptive integration scheme recursively subdivides the triangles until the required precision is achieved. This adaptive integration has an influence on the accuracy, as will be exposed in the benchmark results further on.

Since the electric potential can only be computed up to a constant, the system matrix is deflated to make it invertible. In practice, it consists of imposing the constraint that the integral of the potential over the external layer is 0.

The magnetic field is computed by using the Biot and Savart equation with the Galerkin piecewise linear approximation for the potential [11].

As stated above, given a set of dipole positions and orientations, a set of sensors and a head model defining homogeneous conductivity regions, the M/EEG forward problem produces as output the *leadfield*. OpenMEEG computes such lead fields with the following procedure (see the tutorial in Additional file 1 for a detailed graphical representation of this flowchart):

• assemble the system matrix involving boundary integral operators on the discretized surfaces;

• for a specified set of dipole positions and orientations, assemble a discretized, source-related term;

• solve the linear system relating the two above matrices (providing *V *and $\sigma \frac{\partial V}{\partial n}$ on each interface)

• interpolate the scalp potential (for EEG) or apply the discretized Biot and Savart relation (for MEG).

Mathematical details, as well as the practical usage of OpenMEEG can be found in the Appendix.

### Methods: benchmark comparison study

In order to motivate the use of the OpenMEEG software, we have conducted a set of numerical experiments that compare the accuracy and the robustness of OpenMEEG with alternative M/EEG forward solvers. The comparison in the context of EEG forward modeling is run with the 4 alternative BEM solvers (see [21] for a comparison with FEM). The precision of the MEG forward solver is demonstrated using known analytical properties of the magnetic field when considering sphere models and with two other solvers.

### Publicly available M/EEG forward solvers

Several software projects have the ability to solve the M/EEG forward problems. MNE, BrainStorm, EEGLAB (via the NFT Toolbox), Fieldtrip, Simbio, OpenMEEG and SPM, which shares with Fieldtrip the same M/EEG forward solvers.

Fieldtrip and SPM offer two implementations of the BEM. The first one, called *Dipoli*, was written by Oostendorp [22] and is not open source (only binary files for UNIX systems are available), while the second one, called *BEMCP*, is opensource and was written by Christophe Phillips during his PhD [23]. The *Dipoli *solver implements a linear collocation method with Isolated Skull Approach (ISA) [24], whereas *BEMCP *implements a simple linear collocation method. The *Dipoli *implementation details can be found in [22]. Another implementation with linear collocation (with and without ISA) can be found in the Matlab toolbox called Helsinki BEM [25]. The MNE package written in pure C code by Hämäläinen also offers a linear collocation (with ISA) implementation of the BEM. The Brainstorm toolbox in its latest version uses only sphere models for both EEG and MEG. Recently the EEGLAB toolbox also has provided a package called NFT that is based on a BEM called METU [26]. Finally, the Simbio forward solver also consists of a BEM with linear collocation and ISA.

Commercial software packages are not listed here. However, to our knowledge, commercial products that provide a BEM solver for forward modeling implement a linear collocation method with ISA. This is for example the case of ASA [27]. Note also that beyond the field of brain research, alternative BEM implementation exist. The SCIRun/BioPSE project contains for example a BEM implementation based on [19] that can solve the forward problem of electrocardiography [28].

### Accuracy measures

The accuracy of forward solvers can be assessed for simple geometries such as nested spheres, by comparison with analytical results. The precision of a forward solution is tested with two measures. the RDM (Relative Difference Measure) and the MAG (Magnitude ratio) [29].

The RDM between the forward field given by a numerical solver *g_n _*and the analytical solution *g_a _*is defined as:

$$RDM(g_{\text{n}},g_{\text{a}})=\left\|\frac{g_{n}}{\left\|g_{n}\right\|}-\frac{g_{a}}{\left\|g_{a}\right\|}\right\|\in \left[0,2\right],$$

while the MAG between the two forward fields is defined as:

$$MAG(g_{n},g_{a})=\frac{\left\|g_{n}\right\|}{\left\|g_{a}\right\|}.$$

In both of these expression, the norm is the discrete *ℓ*^2 ^norm over the set of sensor measurements. The closer to 0 (resp. to 1) the RDM (resp. the MAG), the better it is.

### Geometrical models

**Figure 2:**
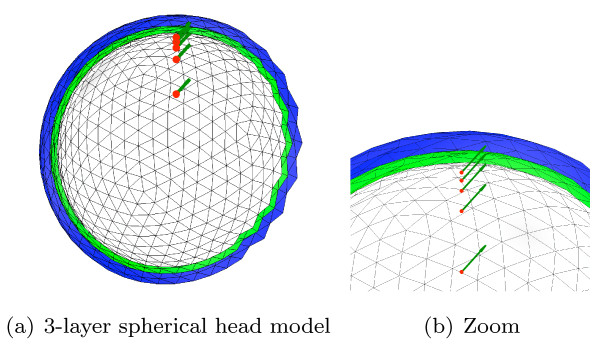
**Head model made of 3 nested regular sphere meshes and 5 dipoles**. Head model made of 3 nested regular sphere meshes with 5 dipoles close to the inner layer.

The comparisons were made both on classic regular sphere meshes as in Figure 2, and on random meshes. A random sphere mesh with unit radius and N vertices is obtained by randomly sampling 10N 3D points, normalizing them, meshing their convex hull and decimating the obtained triangular mesh from 10N to N vertices. This process guarantees an irregular meshing while avoiding flat triangles. The BEM solvers are tested with three nested sphere shells which model the inner and outer skull, and the skin. The radii of the 3 layers are set to 88, 92 and 100, while the conductivities of the 3 homogeneous volumes are set to 1, 1/80 (skull) and 1. In this benchmark, the units are arbitrary, but in practice, units should be expressed with the International System of Units (SI). For each randomly generated head model, it was tested that they were no intersection between each mesh. For every head model, solvers are tested with the same 5 dipoles positioned on the z-axis with orientation (1,0,1) and various distances to the inner layer (cf. Figure 2). As expected, the accuracy of the solvers decreases as this distance gets small.

## Results

### Results: accuracy of the electric potential simulations

The implementations tested for EEG are: OpenMEEG with and without adaptive numerical integration (abbr. OM and OMNA), Simbio (abbr. SB), Helsinki BEM with and without ISA (abbr. HBI and HB), Dipoli (abbr. DP) and BEMCP (abbr. CP). The METU solver was also tested but we were unable to obtain the precisions advertised in [26], so it was decided not to include it in the comparison.

**Figure 3:**
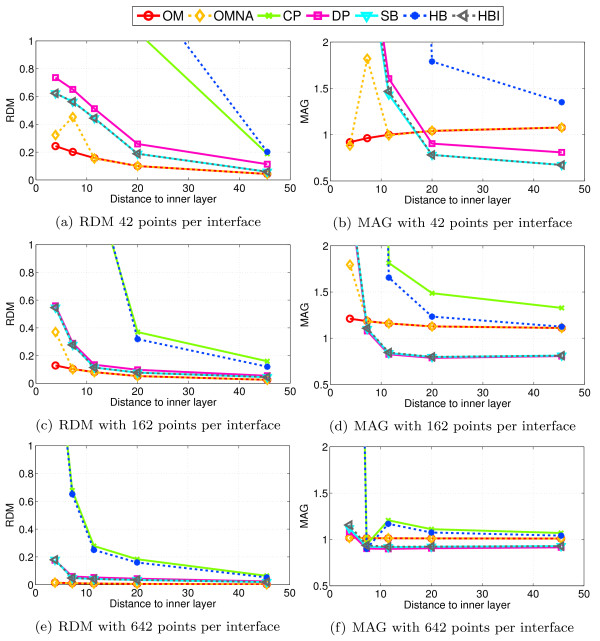
**Accuracy comparison of the different BEM solvers for EEG**. Forward EEG: accuracy comparison of different BEM solvers with three-layers sphere head models. We observe that the Symmetric BEM outperforms the other methods in term of precision.

The results with regular sphere meshes are presented in Figure 3 for 3 different point samplings on each interface. The coarsest sampling has only 42 vertices per interface and 42 EEG electrodes, the intermediate one has 162 points per interface and 162 EEG electrodes, and the finest sampling has 642 points per interface and 642 EEG electrodes. In this case, electrodes are simply located at mesh nodes.

From these simulations it can be observed that:

• HB and CP, that implement a simple linear collocation method, have similar results and are clearly the less precise solvers.

• HBI, SB and DP, that implement a linear collocation method with ISA, have very similar results. SB and HBI are however slightly more accurate than DP.

• OpenMEEG provides the most precise solutions even when no adaptive integration is used. The adaptive integration significantly improves the results, particularly when the meshes are coarsely sampled (42 and 162 vertices per layer).

**Figure 4:**
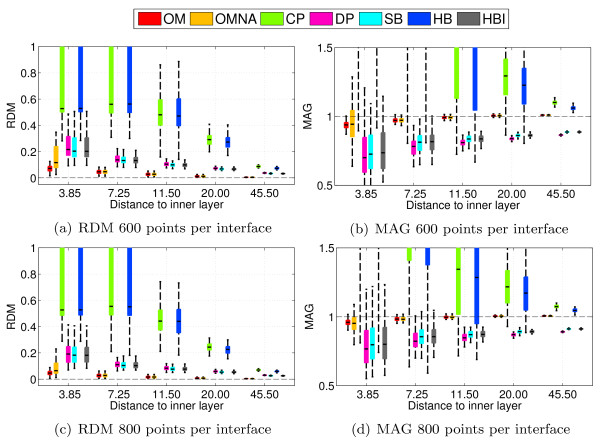
**Accuracy comparison for EEG using random meshes with fixed number of vertices**. Forward EEG: RDM and MAG boxplots obtained on 100 random 3-layers sphere models. Each layer contains 600 or 800 random vertices.

Simulations have also been run on a large number of randomly sampled sphere meshes, in order to compare the robustness of the different solvers. Each result is obtained by running all solvers on 100 random head models. The mean accuracy measures (RDM and MAG) are represented using boxplots, in order to display the variance of the errors. Figure 4 presents the boxplots obtained by running the solvers on random head models with either 600 or 800 vertices per interface. These results lead to the same ranking of methods as those of Figure 3, if the average accuracy is considered. However the variances are also very informative, as they tell us about the precision. It can be observed that OM is not only very accurate, but also very precise because of its very small variance, which is an appreciable feature. The OMNA solver is also accurate but less precise. it has a larger variance. This demonstrates that the adaptive integration makes the solver more robust to irregular meshing. SB and HBI give, as expected, very similar results. One can also observe that the variances observed for CP and HB are significantly larger than for the other solvers, meaning that the collocation based BEM without ISA is very sensitive to irregular meshing.

**Figure 5:**
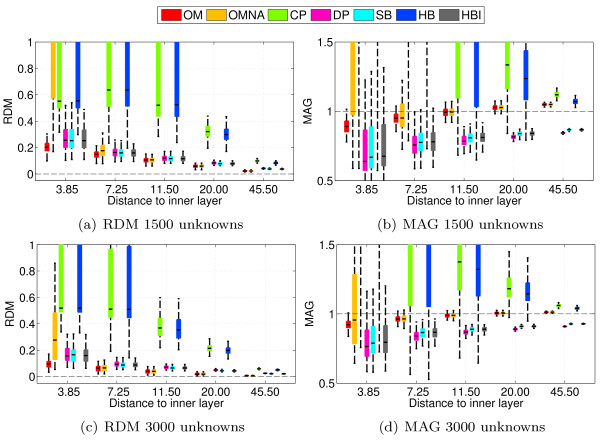
**Accuracy comparison for EEG using random meshes and fixed number of unknowns**. Forward EEG: RDM and MAG boxplots obtained on 100 random 3-layers sphere models. Each forward solution is obtained taking as constraint that the number of unknowns that is estimated is the same for all BEM solvers. Results are presented for 1500 and 3000 unknowns.

As explained in the previous section, OpenMEEG considers as unknowns both the potential at vertices, and the normal current at the centers of the triangles. For a fair comparison with respect to numerical complexity, the previous experiments have been repeated with the constraint that each solver should handle *an equal number of unknowns*. This leads to considering meshes for OpenMEEG with less points than for others solvers. A closed triangular mesh with *n *vertices contains 2*n *- 4 triangles and the normal current is not discretized for the outer layer as it is fixed to be 0. For a three-layer BEM, OpenMEEG therefore has to handle 7*n *- 8 unknowns, while for a standard BEM this number is simply 3 *n*. For a fixed number *n_u _*of unknowns, the number of vertices per layer *n_om _*for OpenMEEG is *n_om _*= (*n_u _*+ 8)/7 while for a standard BEM the number of vertices *n_std _*is *n_std _*= *n_u_*/3. Results with 1500 and 3000 unknowns are presented in Figure 5. It can be observed that OpenMEEG with adaptive integration still outperforms other solvers in term of mean accuracy as well as variance of the results. It is followed by DP, HBI and SB, that give also quite accurate solutions. OpenMEEG without adaptive integration is in this simulation behind DP, HBI and SB but remains more precise than HB and CP.

### Results: accuracy of the magnetic field simulations

We next present results for MEG forward modeling. MEG manufacturers propose 3 kinds of sensors (magnetometers, axial gradiometers, and planar gradiometers), all of which are oriented radially with respect to the helmet.

**Figure 6:**
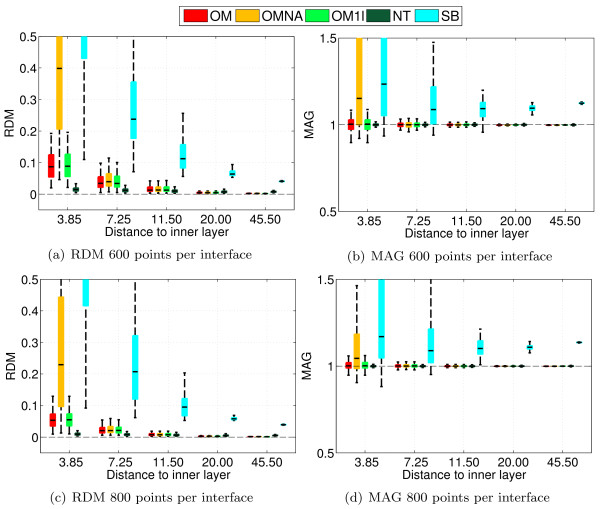
**Accuracy comparison for MEG using random meshes with fixed number of vertices and non-radial magnetometers**. Forward MEG: RDM and MAG boxplots obtained on 100 random sphere models (1 and 3-layers) using non-radial magnetometers. Each layer contains 600 or 800 random vertices.

**Figure 7:**
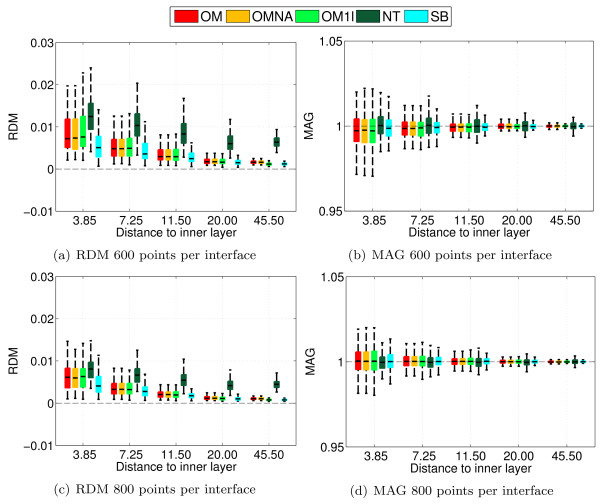
**Accuracy comparison for MEG using random meshes with fixed number of vertices and radial magnetometers**. Forward MEG: RDM and MAG boxplots obtained on 100 random sphere models (1 and 3-layers) using radial magnetometers. Each layer contains 600 or 800 random vertices.

With a nested sphere model, Ohmic volume currents do not contribute to the radial component of the magnetic field [4] (the term containing *σ*Δ*V *in (3) vanishes). The MEG community commonly uses analytical solutions on spheres to compute MEG leadfields although volume currents do have an influence on the magnetic field when considering realistic head models [30]. OpenMEEG (as well as Simbio) uses the previously computed electric potential on all surfaces to compute the contribution of the volume currents to the magnetic field at sensors. These considerations lead to two different setups to validate the MEG forward solutions provided by OpenMEEG. To do so, experiments have been run with two types of sensors. a set of magnetometers all oriented in the Cartesian direction (1, 0, 1) and located at a distance of 120 from the center of the spheres, and a set of magnetometers at the same locations but radially oriented. In these experiments, we compared the analytical results with the solutions given by Simbio, by OpenMEEG with and without adaptive integration (abbr. OM and OMNA). We used a 3-layer model, and also a single layer model (the inner skull) as commonly done in practice. The single layer solution is abbreviated OM1L. The Fieldtrip Toolbox provides a solution to the MEG forward problem on realistic volume conductors which is not based on the Biot and Savart law but Helmholtz's reciprocity principle [31]. This solver proposed by Nolte is abbreviated NT in the comparison results. Figure 6 presents the results with non-radial magnetometers, while Figure 7 presents the results obtained with radial magnetometers. From Figure 6 it can be observed that OpenMEEG provides solutions that are considerably more precise with the adaptive integration method. The results of Simbio and OpenMEEG with adaptative integration are mesh-dependent, whereas Nolte's solver outperforms OpenMEEG and Simbio. In Figure 7, one can notice the correct cancellation of the volume current when the mesh size increases (notice the change of scale on the vertical axis). The OpenMEEG and Simbio solvers take the lead for radially oriented sensors with similar results for both.

### Results: computation speed

**Figure 8:**
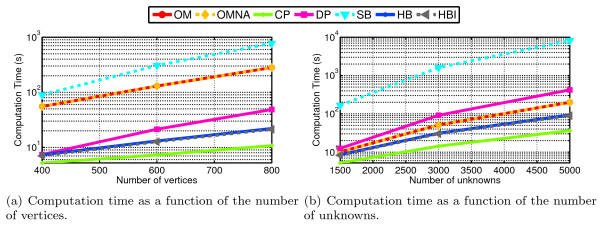
**Computation times of the different BEM solvers for EEG**. Forward EEG: computation times for the different solvers as a function of the number of vertices per layer or the number of unknowns.

We have compared the computation times of EEG forward solvers in two situations. with a fixed number of vertices per layer, and with a fixed number of unknowns. When the number of vertices is fixed, the higher number of unknowns in the symmetric BEM makes the problem size larger. This is confirmed by the results presented in Figure 8(a) where is can be observed that OpenMEEG is slower than all solvers except Simbio. One explanation is that Simbio does not use BLAS/LAPACK for efficient linear algebra but implements its own routines in C code. When the number of unknowns is fixed (cf. Figure 8(b)), the computation time of OpenMEEG is comparable with Dipoli and even slightly lower for highly sampled models. In all cases the collocation methods without ISA (HB and CP) are significantly faster, but their limited accuracy does not make them good candidates for EEG forward modeling. By jointly analyzing Figures 5 and 8(b), one can note that OpenMEEG is the fastest method for a prescribed accuracy.

## The OpenMEEG Software Package

All the tests performed in this paper were made using the version 2.0 of OpenMEEG.

### Licence

OpenMEEG is distributed under the French opensource license CeCILL-B. It is intended to give users the freedom to modify and redistribute the software. It is therefore compatible with popular opensource licenses such as the GPL and BSD licenses. The CeCILL-B license imposes to anybody distributing a software incorporating OpenMEEG the obligation to give credits (by citing the appropriate publications), in order for all contributions to be properly identified and acknowledged. The references to be acknowledged are [7], and the present article.

### Source code

OpenMEEG is implemented in C/C++ with limited external dependencies. It uses the Intel MKL libraries on Windows and ATLAS (BLAS/LAPACK) on Unix systems for fast and accurate linear algebra. A modified version of the MATIO library http://sourceforge.net/projects/matio has been integrated in OpenMEEG for Matlab compatible IOs for vectors and matrices. The source code of OpenMEEG is hosted on the INRIA GForge platform and is accessible to an anonymous user via a public version control system. OpenMEEG binaries and source code are both available from http://openmeeg.gforge.inria.fr.

### Multiplatform

OpenMEEG is available as precompiled binaries for GNU-Linux systems, Mac OS and Windows. OpenMEEG's build and packaging system is based on *CMake*/*CPack *http://www.cmake.org allowing easy development and deployment on all architectures.

### Parallel processing

Compilation of OpenMEEG can be done using advanced features provided by modern compilers. OpenMP is a technology that enables parallel computation at a limited cost in terms of software design. When OpenMEEG is compiled using OpenMP, the numerical integration, on which most of the computation time is spent, can be run in parallel. On a machine with 8 CPUs a standard EEG leadfield is computed up to 6 times faster.

**Figure 9:**
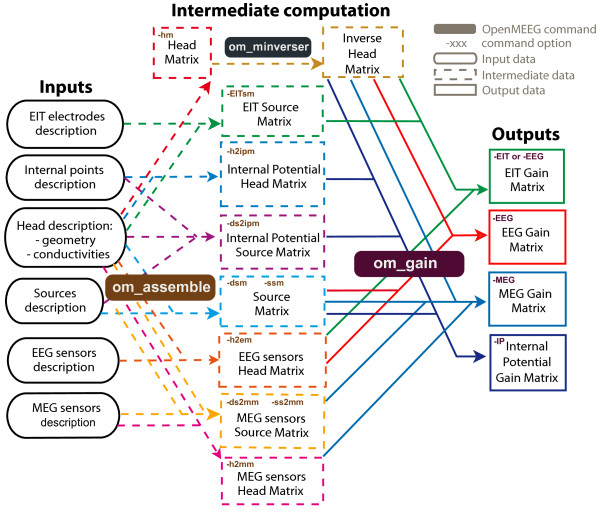
**Pipelines for computing lead fields with OpenMEEG**. Diagram for the low level pipeline for computing MEG and EEG leadfields (a.k.a., gain matrices) using OpenMEEG. To facilitate the understanding of this diagram one can give an example. An EEG gain matrix is obtained with the *om_gain *command using with option -*EEG *taking as input an inverted head matrix, an EEG sensors matrix and a source matrix. The source matrix can be obtained using *om_assemble *taking as input a head model (geometry and conductivities) and a source descriptionfile (option -*dsm *when using isolated dipoles). The inverted head matrix is obtained using *om_minverser *from a head matrix which is obtained using *om_assemble *and the option -*HM *from a head model (geometry and conductivities).

### Testing

Deployment on multiple architectures with heterogenous hardware and software environments requires testing procedures to assess the stability of the solutions provided by compiled binaries. This testing procedure is run through the *CMake*/*CTest *testing software. OpenMEEG test suite guarantees the integrity of the results obtained by MEG, EEG and EIT forward solvers. A part of the tests consists of running OpenMEEG on a 3-layer spherical geometry like the one presented in Figure 2. Outputs are then compared to analytical results to test that the accuracy is not degraded by a modification of the code.

### Integration

Considerable efforts have been made to facilitate the use of OpenMEEG by the M/EEG community. OpenMEEG can be simply invoked via a command line interface, or via higher levels languages. OpenMEEG can also be called from Python or via the Fieldtrip Toolbox, where it has been fully integrated in the M/EEG forward modeling routines.

For sample scripts using Python and Fieldtrip, see Tables 1 and 2.

**Table 1:** OpenMEEG demo script in Python

Python code
Import openmeeg as om

# *Load data*
condFile = 'om_demo.cond'
geomFile = 'om_demo.geom'
dipoleFile = 'cortex.dip'
squidsFile = 'meg_squids.txt'
electrodesFile = 'eeg_electrodes.txt'

geom = om.Geometry()
geom.read(geomFile,condFile)

dipoles = om.Matrix()
dipoles.load(dipoleFile)

squids = om.Sensors()
squids.load(squidsFile)

electrodes = om.Matrix()
electrodes.load(electrodesFile)

# *Compute forward problem*
gaussOrder = 3; # *Integration order*

hm = om.HeadMat(geom,gaussOrder)
hminv = hm.inverse()
dsm = om.DipSourceMat (geom, dipoles, gaussOrder)
ds2 mm = om.DipSource2MEGMat (dipoles, squids).
h2 mm = om.Head2MEGMat (geom, squids)
h2em = om.Head2EEGMat (geom, electrodes).
gain_meg = om.GainMEG (hminv, dsm, h2 mm, ds2mm)
gain_eeg = om.GainEEG (hminv, dsm, h2em)

Demo script for computing MEG and EEG forward problems with OpenMEEG in Python.

**Table 2:** OpenMEEG demo script in Matlab

Matlab code
%% *Structure for BEM volume conduction model*
%% *Each layer mesh is indexed by k*
% *vol.bnd(k).pnt. : vertices for mesh "k"*
% *vol.bnd (k).tri : triangles for mesh "k"*
% *%% Set the conductivities of each domain*
% *vol .cond : conductivities*

%% *EEG electrodes*
*% sens.pnt : locations of electrodes*

*%% Positions of the dipoles*
*% pos :locations of dipoles*

%% *Compute the BEM*
*% choose BEM method (OpenMEEG, BEMCP or Dipoli)*
**cfg.method = 'openmeeg';**
*% Compute the BEM matrix*
vol = ft_prepare_bemmodel(cfg, vol);
**cfg. vol = vol;**
**cfg. grid. pos = pos;**
**cfg. elec = sens;**
*% Compute leadfield*
*% with no orientation constraint*
**lf_openmeeg = ft_prepare_leadfield(cfg);**

Demo script for computing an EEG forward problem with OpenMEEG in Fieldtrip.

### Benchmark

The benchmark presented in the previous section was run within the Fieldtrip environment and its MATLAB source code can be obtained for noncommercial use from the authors. Since SPM uses the same code as Fieldtrip for forward modeling, SPM can now benefit from integration of OpenMEEG. Moreover, the binary format used by OpenMEEG is that of Matlab, by use of the opensource MATIO library.

A sample dataset for M/EEG forward modeling can be downloaded from http://openmeeg.gforge.inria.fr. The sample dataset is provided with scripts that can be run to compute MEG and EEG leadfields on a realistic 3-layer model.

### Documentation

A tutorial for OpenMEEG is available on the web site, and it is briefly summarised in Additional file 1.The tutorial describes the low-level interface and details the different steps to be followed when computing M/EEG lead fields (Figure 9). Developper documentation can be generated via doxygen http://www.stack.nl/~dimitri/doxygen/.

## Conclusion

In this paper, the OpenMEEG software project has been detailed, from the mathematical grounds of the symmetric BEM to more practical aspects.

The relevance of the OpenMEEG solver for quasistatic bioelectromagnetics has been demonstrated by a benchmark incorporating many alternative solvers in the context of M/EEG forward modeling. According to the results of this simulation study, OpenMEEG outperforms all the alternative solvers tested. By providing state-of-the-art solutions for both EEG and MEG forward problems, OpenMEEG enables the combined use of these two complementary modalities.

It should be mentioned that OpenMEEG is being used for many problems in the field of quasistatic bioelectromagnetics, including Electrical Impedance Tomography, Intracranial electric potentials, Functional Electrical Stimulation and Cortical Mapping. This wide range of application domains, as well as its integration into high-level languages make OpenMEEG unique and particularly valuable for basic and clinical research purposes.

## Competing interests

The authors declare that they have no competing interests.

## Authors' contributions

MC with TP and some other colleagues are at the origin of the mathematical developments of the symmetric BEM. AG designed most of the comparison framework detailed in this paper and carried it out. EO carried out the comparison for MEG. All the authors are the current main developpers of the OpenMEEG software package, with AG and TP playing the role of maintainers. AG led the integration effort with other toolboxes. All coauthors participated in writing the paper and approved the final manuscript.

## Appendix

### The symmetric BEM

In early numerical experiments to compare a Boundary Element and a Finite Element Method (FEM) for forward electroencephalography, we found a superior accuracy of the FEM [21]. This triggered a quest to improve the precision of Boundary Element Methods and led us to study the extended Green representation theorem [6]. We proposed a common formalism for the integral formulations of the forward EEG problem, and derived three different Boundary Element Methods within the same framework [7]. In this section we recall the mathematical background of Boundary Element Methods, and present both the double-layer BEM, which is the most widespread method, and the symmetric BEM, which is a new formulation.

#### Green Representation

A fundamental result in potential theory shows that a harmonic function (i.e., such that Δ*u *= 0) is uniquely determined within a domain Ω from its value on the boundary ∂Ω (Dirichlet condition), or the value of its normal derivative (Neumann condition). The Green Representation Theorem gives an explicit representation of a piecewise-harmonic function as a combination of boundary integrals of its jumps and the jumps of its normal derivative across interfaces. Before stating this theorem, some notation must be defined.

• The restriction of a function *f *to a surface *S_j _*is indicated by *f_sj_*.

• The functions $f_{S_{j}}^{-}$ and $f_{S_{j}}^{+}$ represent the interior and exterior limits of *f *on *S_j_*:

$$\text{for }r\in S_{j},\;f_{S_{j}}^{\pm }(r)=\underset{\alpha \to 0^{\pm }}{\lim}f(r+\alpha n).$$

• The jump of a function *f *across *S_j _*is denoted by:

$${\left[f\right]}_{S_{j}}=f_{S_{j}}^{-}-f_{S_{j}}^{+},$$

• ∂_**n**_*V *= **n**·∇*V *denotes the partial derivative of *V *in the direction of a unit vector **n**,

• The function $G(r)=\frac{1}{4\pi \left\|r\right\|}$ is the fundamental solution of the Laplacian in ℝ^3^, such that -Δ*G *= *δ*_0_.

Consider an open region Ω and a function *u *such that Δ*u *= 0 in Ω and in ℝ^3^\Ω (but not necessarily continuous across ∂Ω). The Green Representation Theorem states that, for a point **r **belonging to ∂Ω,

(4) $$\frac{u^{-}(r)+u^{+}(r)}{2}=-\int_{\partial \Omega }\left[u]\partial_{n^{\prime }}G(r-r^{\prime })ds(r^{\prime }\right)+\int_{\partial \Omega }\left[\partial_{n^{\prime }}u]G(r-r^{\prime })ds(r^{\prime }\right).$$

This representation also holds for the head model in Figure 1, when Ω is the union of disjoint open sets: Ω = Ω_1 _⋃ Ω_2 _⋃ ... Ω*_N_*, with ∂Ω = *S*_1 _⋃ *S*_2 _⋃ ... *S_N_*. If *u *is harmonic in each Ω*_i_*, for **r **∈ *S_i_*,

(5) $$\frac{u^{-}(r)+u^{+}(r)}{2}=-\sum_{j=1}^{N}\left(\int_{S_{j}}{\left[u\right]}_{S_{j}}\partial_{n^{\prime }}G(r-r^{\prime })ds(r^{\prime })+\int_{S_{j}}{\left[\partial_{n^{\prime }}u\right]}_{S_{j}}G(r-r^{\prime })ds(r^{\prime })\right)$$

The notation is simplified by introducing two integral operators, which map a scalar function *f *on ∂Ω to another scalar function on ∂Ω: the "double-layer" operator $\left(Df)(r)=\int_{\partial \Omega }\partial_{n^{\prime }}G(r-r^{\prime })f(r^{\prime })\text{d}s(r^{\prime }\right)$, and the "single-layer" operators $\left(Sf)(r)=\int_{\partial \Omega }G(r-r^{\prime })f(r^{\prime })\text{d}s(r^{\prime }\right)$. For an operator D, its restriction $D_{ij}$ maps a function of *S_j _*to a function of *S_i_*.

An extension of the Green Representation Theorem represents the directional derivative of a harmonic function as a combination of boundary integrals of higher order. This requires two more integral operators: the adjoint $D^{*}$ of the double-layer operator, and a hyper-singular operator N defined by $\left(Nf)(r)=\int_{\partial \Omega }\partial_{n,n^{\prime }}G(r-r^{\prime })f(r^{\prime })\text{d}s(r^{\prime }\right)$ if **r **is a point of *S_i_*,

(6) $$-\frac{\partial_{n}u^{-}(r)+\partial_{n}u^{+}(r)}{2}=N[u]-D^{*}[\partial_{n}u],$$

where $D^{*}$ is the adjoint of the operator D. The Geselowitz formula exploits only the first boundary integral representation equation (5), while it is possible to exploit both (5) and (6). Thus three Boundary Element Methods can be derived within a unified setting: a BEM involving only single-layer potentials, a BEM involving only double-layer potentials, and a symmetric BEM combining single- and double-layer potentials [7]. We concentrate hereforth on the double-layer and on the symmetric BEMs.

### The double-layer BEM

To apply the representation theorem to the forward problem of EEG, a harmonic function must be produced, which relates the potential and the sources. Decomposing the source term as *f *= ∑*_i _f_i _*where the support of each *f_i _*lies inside Ω*_i_*, consider $v_{\Omega_{i}}$ such that $\Delta v_{\Omega_{i}}=f_{i}$ holds in all ℝ^3^. The function $v_{d}=\sum_{i=1}^{N}v_{\Omega_{i}}$ satisfies Δ*v_d _*= *f *and is continuous across each surface *S_i_*, as well as its normal derivative ∂**_n_***v_d_*. The function *u *= *σV *- *v_d _*is a harmonic function in Ω, to which (5) can be applied. Since ${\left[u\right]}_{S_{i}}=(\sigma_{i}-\sigma_{i+1})V_{S_{i}}$ and [∂**_n_***u*] = 0, we obtain, on each surface *S_i_*,

(7) $$\frac{\sigma_{i}+\sigma_{i+1}}{2}V_{S_{i}}+\sum_{j=1}^{N}(\sigma_{j}-\sigma_{j+1})D_{ij}V_{S_{j}}=v_{d}.$$

The above formula was established by Geselowitz [5], and was the only one used to model electroencephalography or electrocardiography, until recently, when [7] showed the diversity of BEMs that can be derived. This classical BEM is called a double-layer BEM because it only involves the double-layer operator D.

### The symmetric BEM

The originality of the symmetric Boundary Element Method is to consider a different piecewise harmonic function for each domain: the function *u*Ω*_i _*equal to $V-\frac{v_{\Omega_{i}}}{\sigma_{i}}$ within Ω*_i _*and to $-\frac{v_{\Omega_{i}}}{\sigma_{i}}$ outside of Ω*_i_*. This $u_{\Omega_{i}}$ is indeed harmonic in ℝ^3^\∂Ω*_i_*, and the representation equations (5) and (6) can be applied, leading to a system of integral equations involving two types of unknowns: the potential *V_i _*and the normal current (σ∂**_n _***V*)*_i _*on each interface.

The surfaces are represented by triangular meshes. To fix ideas, consider a three-layer geometrical model for the head. Conductivities of each domain are respectively denoted *σ*_1_, *σ*_2 _and *σ*_3_. The surfaces enclosing these homogeneous conductivity regions are denoted *S*_1 _(inner skull boundary), *S*_2 _(skull-scalp interface) and *S*_3 _(scalp-air interface). Denoting $\psi_{i}^{\left(k\right)}$ the P0 function associated to triangle *i *on surface *S_k_*, and $\phi_{j}^{\left(l\right)}$ the P1 function associated to node *j *on surface *S_l_*, the potential *V *on surface *S_k _*is approximated as $V_{S_{k}}(r)=\sum_{i}x_{i}^{\left(k\right)}\phi_{i}^{\left(k\right)}\left(r\right)$, while *p *= *σ*∂**_n _***V *on surface *S_k _*is approximated by $p_{S_{k}}(r)=\sum_{i}y_{i}^{\left(k\right)}\psi_{i}^{\left(k\right)}(r)$. As an illustration, considering the source term to reside in the brain compartment Ω_1_, the variables ${\left(x_{k}\right)}_{i}=x_{i}^{\left(k\right)}$ and ${\left(y_{k}\right)}_{i}=y_{i}^{\left(k\right)}$ satisfy the linear system:

(8) $$\left[\begin{array}{ccccc} {\hat{\sigma }}_{12}{\text{N}}_{11} & -2{\text{D}}_{11}^{*} & -\sigma_{2}{\text{N}}_{12} & {\text{D}}_{12}^{*} & 0\\ -2{\text{D}}_{11} & {\overset{⌣}{\sigma }}_{12}{\text{S}}_{11} & {\text{D}}_{12} & -\sigma_{2}^{-1}{\text{S}}_{12} & 0\\ -\sigma_{2}{\text{N}}_{21} & {\text{D}}_{21}^{*} & {\hat{\sigma }}_{23}{\text{N}}_{22} & -2{\text{D}}_{22}^{*} & -\sigma_{3}{\text{N}}_{23}\\ {\text{D}}_{21} & -\sigma_{2}^{-1}{\text{S}}_{21} & -2{\text{D}}_{\text{22}} & {\overset{⌣}{\sigma }}_{23}{\text{S}}_{22} & {\text{D}}_{23}\\ 0 & 0 & -\sigma_{3}{\text{N}}_{32} & {\text{D}}_{32}^{*} & \sigma_{3}{\text{N}}_{33} \end{array}\right]\;\left[\begin{array}{c} x_{1}\\ y_{\text{1}}\\ x_{\text{2}}\\ y_{\text{2}}\\ x_{\text{3}} \end{array}\right]=\left[\begin{array}{c} b_{\text{1}}\\ c_{\text{1}}\\ 0\\ 0\\ 0 \end{array}\right]$$

where ${\hat{\sigma }}_{ij}$ (resp.${\overset{⌣}{\sigma }}_{ij}$) is defined as *σ_i _*+ *σ_j _*(resp. $\sigma_{i}^{-1}+\sigma_{j}^{-1}$) and where **b**_1 _and **c**_1 _are the coefficients of the P0 (resp. P1) boundary element decomposition of the source term $\partial_{n}v_{\Omega_{1}}$ (resp. $-\sigma_{1}^{-1}v_{\Omega_{1}}$).

Blocks N*_ij _*and D*_ij _*map a potential *V_j _*on *S_j _*to a function defined on *S_i_*. Block *S_ij _*maps a normal current *p_j _*on *S_j _*to a function defined on *S_i_*. The resulting matrix is block-diagonal, and symmetric, hence the name "symmetric BEM".

The magnetic field is computed from the electric field and the primary source distribution using the Biot and Savart equation (3), as proposed by Ferguson, Zhang and Stroink [11].

In summary, the symmetric BEM introduces an additional unknown into the problem. the normal current, and uses an additional set of representation equations linking the normal current and the potential. The symmetric BEM departs from the double-layer BEM in several ways:

• the normal current to each surface is explicitely modeled;

• only the surfaces which bound a common compartment have an interaction (whence the blocks of zeros in the matrix);

• only the surfaces which bound a compartment containing sources have a source term;

• the matrix is symmetric;

• the matrix is larger for a given head model.

## Supplementary Material

Additional file 1 **OpenMEEG: Hands-on tutorial**. Click here for file
