# Supplementary material for: OpenMEEG: opensource software for quasistatic bioelectromagnetics
Source: Biomed Eng Online. 2010 Sep 6;9:45. doi: 10.1186/1475-925X-9-45 (PMC2949879; doi:10.1186/1475-925X-9-45)
Supplement: Additional file 1 — OpenMEEG: Hands-on tutorial. [file 1475-925X-9-45-S1.PDF]

OpenMEEG: Hands-on tutorial

This tutorial depicts briefly OpenMEEG's different file formats and commands. It then proceeds in giving some examples of common tasks that can be achieved with OpenMEEG.

1 OpenMEEG file formats

OpenMEEG handles several file formats corresponding to several type of objects: vectors, matrices, head geometries, meshes, dipoles, conductivities.

1.1 Vectors and matrices

By default matrices and vectors are stored on disk using a MATLAB file format. Symmetric matrices which are not directly representable in the MATLAB format are represented as a MATLAB struct. Other vector/matrices file formats are also supported. Forcing a specific file format is achieved by specifying the proper file extension. Matlab extension is `.mat`. Other useful file formats are ASCII (extension `.txt`) which generates human readable files, BrainVisa[1] texture file format (extension `.tex`) and OpenMEEG's own binary file format (extension `.bin`) which is available solely for backward compatibility and should be considered as deprecated (as it is subsumed by the MATLAB file format).

1.2 Geometrical model, mesh and conductivity files

OpenMEEG geometrical models are provided through several files. Note that OpenMEEG considers SI units (i.e. point coordinates should be expressed in meters (m), conductivities in S/m, etc). The toplevel file (generally ending with the extension `.geom`) assembles various interface descriptions to build *Domains* corresponding to head tissues. Empty lines or lines beginning with `#` are non-significant. The file must start with a special comment line which allows its identification (see example in Figure 1).
Geometrical models globally contain 2 sections.

The first section specifies the meshes of the interfaces between tissues. It is introduced by the keyword `Interfaces` followed by the number of such meshes followed by the keyword `Mesh` (which gives the type of the mesh description, `Mesh` being currently the only possibility). This specification is then followed by the

[1] http://www.brainvisa.info

names of – mesh – files describing each of the interfaces. Each interface is assigned an index from its order in the list. These indices start at 1.

The second section describes the head tissues and is introduced by the keyword `Domains` followed by the number of such domains. Each domain is then depicted, one domain per line, by the keyword `Domain` followed by the domain name (which will serve for identification and will also appear in the conductivity description) followed by a list of integers. These integer are the indices of the mesh files (as depicted in previous paragraph). They are affected by a + or `-` sign depending on whether the domain is outside or inside the corresponding interface (as defined by the outward normal of the interface). See Figure 1 for a detailed example.

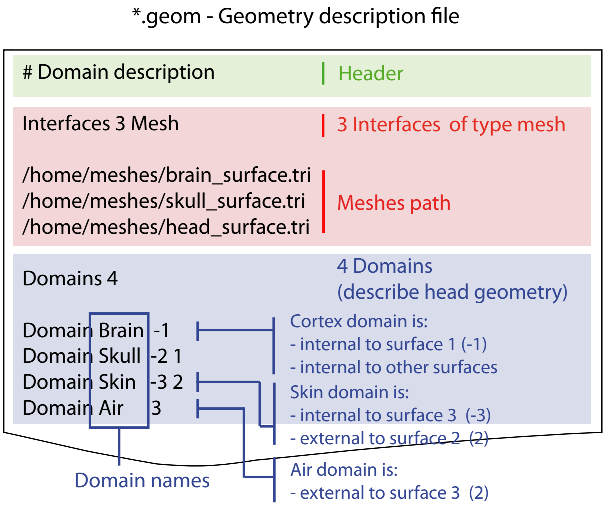

Figure 1: Sample geometry file.

Mesh files (generally ending with the `.tri` extension) follow the BrainVisa[2] file format for meshes. These files contain two sections. Each section is introduced by the character `-` appearing at the beginning of the line followed by a space followed by either one number (first section) or three times the same number (second section).

The first section contains a list of points with associated normals. The number on the line introducing the section is the number of points. Each following line corresponds to a single point. Its coordinates are the three first numbers appearing on the line. The normal corresponds to the following three numbers. Each point is assigned an index (starting at 0) corresponding to its order of appearance in the list.

[2]Please note that FreeSurfer also provides `.tri` files. Those are not handled by OpenMEEG yet.

The second section contains the triangles of the mesh. The number (repeated three times) in the section delimiter corresponds to the number of triangles. Each triangle is depicted by a sequence of three integers corresponding to the indices of the points assigned as explained in the previous paragraph.

The following small example describes a very simple mesh containing 4 points and 4 triangles.

```
- 4
0 0 0 -0.5773 -0.5773 -0.5773
1 0 0 1 0 0
0 1 0 0 1 0
0 0 1 0 0 1
- 4 4 4
0 1 2
0 1 3
0 2 3
1 2 3
```

The interface meshes are required to be closed in order for the Boundary Element Method to function correctly. This is also necessary for the source meshes when computing forward solutions using surfacic source models (see below). Moreover, the interface meshes must not intersect each other. Non-intersection can be checked with the command `om_check_geom`. The command `om_mesh_info` applied to a mesh provides its number of points, of triangles, minimum and maximum triangle area, and also its Euler characteristic. The Euler characteristic of a closed mesh of genus 0 (homotopic to a sphere) is equal to 2. The Euler characteristic gives an indication if a mesh is likely to be closed or not.

A **conductivity file** (generally ending with the extension `.cond`) is a simple ASCII file that contains associations between tissue names and conductivity values. Associations are provided one per line. Empty lines or lines beginning with `#` are non-significant. The file must start with a special comment line which allows its identification. Figure 2 provides an example conductivity file corresponding to the geometry file of Figure 1.

Note that the tissue names are the ones appearing in the *Domain description* section of the file depicting the geometrical model.

1.3 Source description

Sources may be represented either by a surfacic distribution of dipoles, or by isolated dipoles.

A **surfacic distribution** can be defined by a mesh that supports the dipoles. The dipole orientations are then constrained to the normal direction to the mesh and the moment amplitude is modelled as continuous across the mesh (piecewise linear). Source values are defined at the mesh vertices.

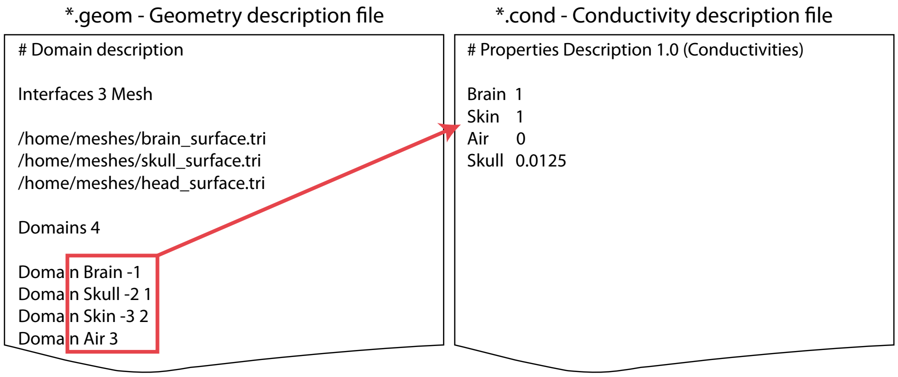

Figure 2: Sample conductivity file and its correspondance with the geometry file.

Isolated dipoles are defined by a simple ASCII file as shown in Figure 3.

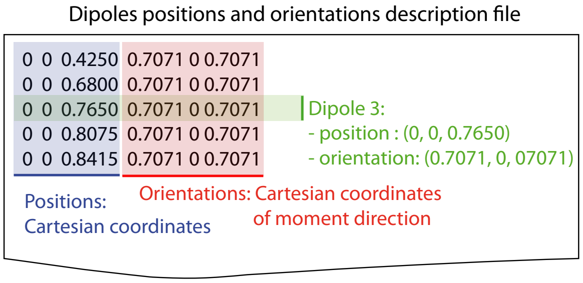

Figure 3: Sample source description for isolated dipoles.

1.4 Sensor description

EEG sensors are supposed pointlike and are represented by their names and positions (x,y,z coordinates). MEG sensors are represented by their names, positions, orientations and weighting coefficients. Gradiometers can hence be defined by the linear combination of two magnetometers with opposite weights. These parameters are defined by an ASCII file as shown in Figure 4.

2 OpenMEEG from the command line

This section reviews the main OpenMEEG command line tools. The general syntax and main options of each command is briefly provided. Full details are available in OpenMEEG documentation and 5 provides a flowchart summarizing the use of these commands. To facilitate the understanding of this diagram one can give an example: An EEG gain matrix is obtained with the *om_gain* command using with option *-EEG* taking as input an inverted head matrix, an EEG sensors matrix and a source matrix. The source matrix

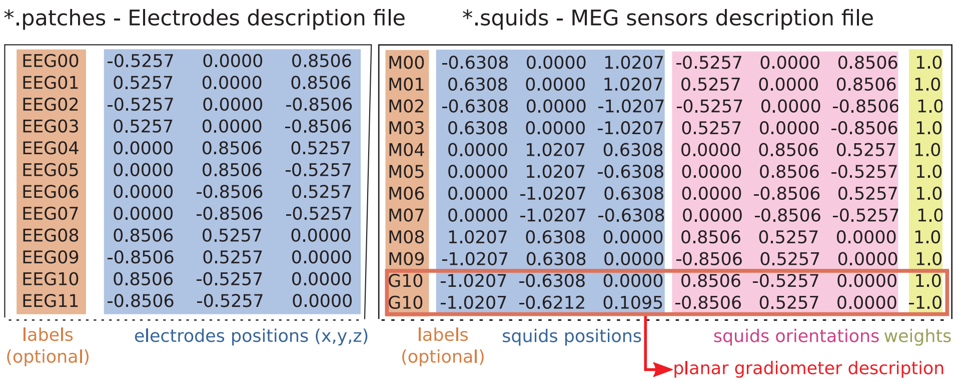

Figure 4: Sample sensors description for EEG and MEG.

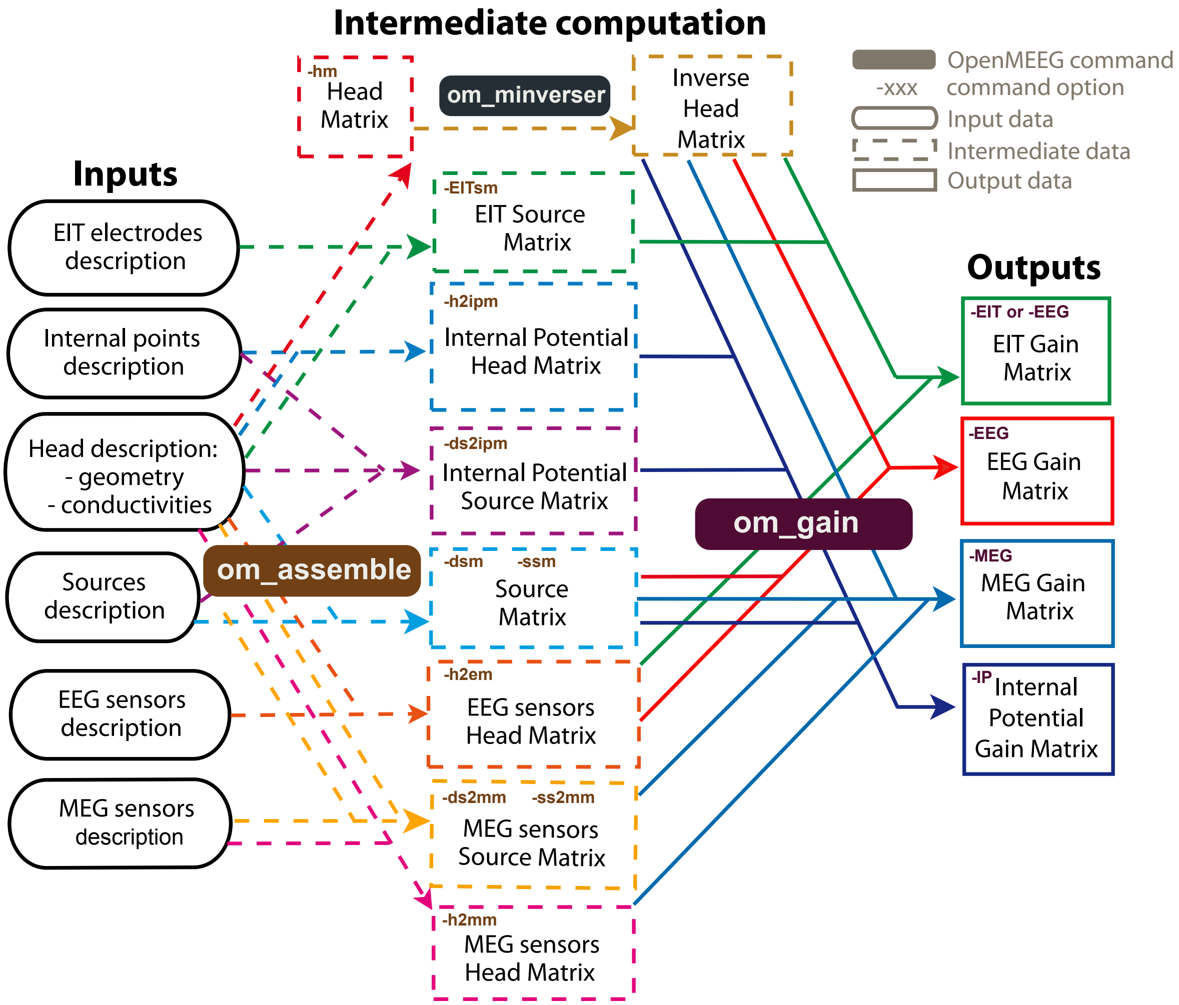

Figure 5: Diagram for the low level pipeline for computing MEG and EEG leadfields (a.k.a., gain matrices) using OpenMEEG.

can be obtained using *om_assemble* taking as input a head model (geometry and conductivities) and a source description file (option *-dsm* when using isolated dipoles). The inverted head matrix is obtained using *om_minverser* from a head matrix which is obtained using *om_assemble* and the option *-HM* from a head model (geometry and conductivities).

In this section, command names are in `red`*, options are in* `green` *and produced files are shown in* `blue`.

2.1 om_assemble

General syntax: `om_assemble Option Parameters Matrix`

This program assembles the different matrices that will be used in later stages. It uses the head description, the sources and the sensors information. `Option` selects the type of matrice to assemble. `Parameters` depends on the specific option `Option`. Except if otherwise noted, it takes the form:

`subject.geom subject.cond OptParam`

where subject.geom and subject.cond are files describing respectively the geometrical model and the conductivities of the head (see above for a short description of these files). `OptParam` depends on the actual `Option`. `Matrix` is the name of the output file containing the computed matrix.
We now detail the possible `Options` (with their abbreviated versions given in parentheses), allowing to define various matrices to assemble:

General options for `om_assemble`

`-help` (`-h`,`--help`): summarizes all possible options.

Head modelling options for `om_assemble` produce matrices linked to the propagation of electrical signals in the head.

`-HeadMat` (`-HM`, `-hm`): `om_assemble` computes the Head matrix for Symmetric BEM (left-hand side of the linear system presented in the appendix). This matrix corresponds to the propagation of electrical signals within the head. There is no `OptParam` in this case.

Source modelling options for `om_assemble` compute the source matrix for Symmetric BEM (right-hand side of the linear system). This matrix maps the representation of $\mathbf{J}^{\mathrm{p}}$ to its associated electric potential in an infinite medium (v_{Ω_1} in previous Appendix). Different options exist for the 2 types of source models:

`-SurfSourceMat` (`-SSM`, `-ssm`): should be used for continuous surfacic distributions of dipoles. `OptParam` is a file containing a mesh that describes the surface.

-DipSourceMat (-DSM, -dsm): should be used when considering several isolated dipoles. This model is the most commonly used and should be used by default even if the dipoles correspond to the vertices of a cortical mesh. OptParam is a file containing the dipolar source description.

Sensor modelling options for om_assemble compute matrices that integrate source information and computed potentials to provide the actual solution of the forward problem. The situation is slightly different for EEG, which only needs to compute the electric potential, and for MEG, which depends both on the electric potential and on the sources:

-Head2EEGMat (-H2EM, -h2em): om_assemble computes the interpolation matrix that maps potentials computed on the scalp to EEG sensors. OptParam is a file describing the EEG sensor positions.

-Head2MEGMat (-H2MM, -h2mm): om_assemble computes the contribution of Ohmic currents to the MEG sensors. OptParam is a file describing the SQUIDS geometries and characteristics.

-Head2InternalPotMat (-H2IPM, -h2ipm): om_assemble computes the matrix that allows the computation of potentials at internal positions from potentials and normal currents on head interfaces, as computed by the symmetric BEM.

-SurfSource2MEGMat (-SS2MM, -ss2mm): om_assemble computes the source contribution to the MEG sensors using the same source model as the one used for the option -SurfSourceMat, i.e. surfacic distribution of dipoles. For this option, OptParam takes the form:

```
mesh squids
```

where `mesh` contains a mesh describing the source surface and `squids` is a file describing the SQUIDS geometries and characteristics.

-DipSource2MEGMat (-DS2MM, -ds2mm): om_assemble computes the source contribution to the MEG sensors using the same source model as the one used for the option -DipSourceMat, i.e. isolated dipoles. For this option, OptParam takes the form:

```
dipoles squids
```

where `dipoles` contains the dipolar source description and `squids` is a file describing the SQUIDS geometries and characteristics.

-DipSource2InternalPotMat (-DS2IPM, -ds2ipm): om_assemble computes the source contribution to the chosen internal points. It gives the potential due to isolated dipoles, as if the medium were infinite. For this option, OptParam takes the form:

dipoles internalPoints

where dipoles contains the dipolar source description and internalPoints is a file describing the points locations.

EIT options for om_assemble

-EITSourceMat (-EITSM, -EITsm,): om_assemble computes the right-hand side for scalp current injection. This usage of om_assemble outputs the right-hand side vector for a given set of EIT electrode. For this option, OptParam is a file describing the EIT electrode positions.

2.2 om_minverser

General syntax: om_minverser HeadMat HeadMatInv

This program is used to invert the symmetric matrix as provided by the command om_assemble with the option -HeadMat. This command has only one option.

-help (-h,--help): summarizes the usage of om_minverser.

2.3 om_gain

General syntax: om_gain Option HeadMatInv Parameters GainMatrix

This command computes the gain matrix by multiplying together matrices obtained previously (e.g. HeadMatInv is the matrix computed using om_minverser). The resulting gain matrix is stored in the file GainMatrix. Option selects the type of matrice to build. Parameters depend on the specific option Option. Possible options are:

General options

-help (-h,--help): summarizes the usage of om_gain for all its possible options.

Gain matrix type options select the type of gain matrix to be computed by om_gain.

-EEG : allows to compute an EEG gain matrix.

Parameters are then:

HeadMatInv SourceMat Head2EEGMat

SourceMat is the matrix obtained using om_assemble with either of the options -SurfSourceMat or -DipSourceMat, depending on the source model. Head2EEGMat is the matrix obtained using om_assemble with the option -Head2EEGMat.

The -EEG option can also be used to compute an EIT gain matrix (see below for the signification of the parameters in this case).

-EIT : allows to compute an EIT gain matrix. This option is actually an alias for the -EEG option in newer versions. For OpenMEEG versions 2.0 and below please use the -EEG option.

Parameters are thus:

`HeadMatInv SourceMat Head2EITMat`

`SourceMat` should contain the output of the -EITsource option of om_assemble. Multiplying the EIT gain matrix by the vector of applied currents at each EIT electrode yields the simulated potential on the EEG electrodes. The applied current on the EIT electrodes should sum to zero. The result is stored in the file `Head2EITMat`

-MEG : allows to compute an MEG gain matrix.

Parameters are then:

`HeadMatInv SourceMat Head2MEGMat Source2MEGMat`

`SourceMat` is the matrix obtained using om_assemble with either of the options -SurfSourceMat or -DipSourceMat, depending on the source model. `Head2MEGMat` is the matrix obtained using om_assemble with the option -HeadMEEGMat. `Source2MEGMat` is the matrix obtained using om_assemble with either of the options -SurfSource2MEGMat or -DipSource2MEGMat, depending on the source model.

-InternalPotential : allows to compute an internal potential gain matrix for sensors within the volume.

Parameters are then:

`HeadMatInv SourceMat Head2InternalPotMat Source2InternalPotMat`

`Head2InternalPotMat` and `Source2InternalPotMat` are respectivelly obtained using om_assemble with option -Head2InternalPotMat and -DipSource2InternalPotMat.

3 Examples

Assuming a head model represented by the geometry file `head.geom` and the conductivity file `head.cond` and EEG sensors detailed in a file `head.eegsensors`. Computing the EEG gain matrix for sources distributed on the surface represented by the file `sources.tri` is done via the following set of commands:

```
om_assemble -HeadMat head.geom head.cond head.hm
om_assemble -SSM head.geom head.cond sources.tri head.ssm
om_assemble -h2em head.geom head.cond head.eegsensors head.h2em
om_minverser head.hm head.hm_inv
om_gain -EEG head.hm_inv head.ssm head.h2em head.gain
```

Considering now isolated dipolar sources detailed in the file `sources.dip` with MEG sensors depicted in the file `head.squids`. Using the same head model, the MEG gain matrix is obtained via the following set of commands:

```
om_assemble -HeadMat head.geom head.cond head.hm
om_assemble -DSM head.geom head.cond sources.dip head.dsm
om_assemble -h2mm head.geom head.cond head.squids head.h2mm
om_assemble -ds2mm sources.dip head.squids head.ds2mm
om_minverser head.hm head.hm_inv
om_gain -MEG head.hm_inv head.dsm head.h2mm head.ds2mm head.gain
```
